# TAOK3 limits age-associated inflammation by negatively modulating macrophage differentiation and their production of TNFα

**DOI:** 10.1186/s12979-023-00350-y

**Published:** 2023-07-03

**Authors:** Alexandre Poirier, Chenyue Wu, Ana Maria Hincapie, Zuzet Martinez-Cordova, Belma Melda Abidin, Michel L. Tremblay

**Affiliations:** 1 Rosalind and Morris Goodman Cancer Institute, McGill University, Montréal, Québec Canada; 2 Faculty of Medicine and Health Sciences, Division of Experimental Medicine, McGill University, Montréal, Québec Canada; 3 Department of Microbiology and Immunology, McGill University, Montréal, Québec Canada; 4 Department of Biochemistry, McGill University, Montréal, Québec Canada; 5 Faculty of Medicine, McGill University, Montréal, Québec Canada; 6 McGill University, Rosalind and Morris Goodman Cancer Institute, 1160 Pine Avenue West, Montréal, Québec H3A 1A3 Canada

**Keywords:** Inflammaging, Macrophages, TAOK3, Pro-inflammatory cytokines, Chemotaxis

## Abstract

**Background:**

Human aging is characterized by a state of chronic inflammation, termed inflammaging, for which the causes are incompletely understood. It is known, however, that macrophages play a driving role in establishing inflammaging by promoting pro-inflammatory rather than anti-inflammatory responses. Numerous genetic and environmental risk factors have been implicated with inflammaging, most of which are directly linked to pro-inflammatory mediators IL-6, IL1Ra, and TNFα. Genes involved in the signaling and production of those molecules have also been highlighted as essential contributors. TAOK3 is a serine/threonine kinase of the STE-20 kinase family that has been associated with an increased risk of developing auto-immune conditions in several genome-wide association studies (GWAS). Yet, the functional role of TAOK3 in inflammation has remained unexplored.

**Results:**

We found that mice deficient in the serine/Threonine kinase Taok3 developed severe inflammatory disorders with age, which was more pronounced in female animals. Further analyses revealed a drastic shift from lymphoid to myeloid cells in the spleens of those aged mice. This shift was accompanied by hematopoietic progenitor cells skewing in Taok3^−/−^ mice that favored myeloid lineage commitment. Finally, we identified that the kinase activity of the enzyme plays a vital role in limiting the establishment of proinflammatory responses in macrophages.

**Conclusions:**

Essentially, Taok3 deficiency promotes the accumulation of monocytes in the periphery and their adoption of a pro-inflammatory phenotype. These findings illustrate the role of Taok3 in age-related inflammation and highlight the importance of genetic risk factors in this condition.

**Supplementary Information:**

The online version contains supplementary material available at 10.1186/s12979-023-00350-y.

## Background

Age-related inflammation, or inflammaging, is a common phenomenon characterized by increased levels of pro-inflammatory factors in the blood [1]. Contrary to acute inflammation and its role in immunity, chronic inflammation is highly detrimental to general health. It is a central risk factor for numerous age-related morbidities such as cardiovascular disease, diabetes, cancer, and kidney disease [1]. Fundamentally, chronic inflammation is hallmarked by a complex disbalance between pro-and anti-inflammatory responses that, in the case of inflammaging, are predominantly controlled by macrophages [2, 3]. Besides their role in apoptotic cell clearance and innate immunity, macrophages are the most potent producers of TNFα, a pro-inflammatory cytokine involved in numerous inflammatory and auto-immune disorders [4]. Systemic lupus erythematosus (SLE) and rheumatoid arthritis (RA) are such diseases and have been found to affect females disproportionally [5]. Sex-based associations remain undetermined in inflammaging, but emerging reports suggest such differences [5, 6]. Thus far, the most important genetic factors for inflammaging are polymorphism in IL-6 [7], its receptor IL-6R [8], and IL-1RN (IL1Ra antagonist gene) [9].

Thousand-and-one (TAO) kinases belong to a subfamily of Ste20-like proteins that can act as MAP4Ks to phosphorylate MAP3Ks [10–13]. The TAO kinase family members have been shown to share kinase domain resemblance and substrate specificity with conventional MAP4K enzymes [14, 15]. The function of those enzymes is involved in immune and inflammatory disorders such as RA and SLE [16]. To our knowledge, it remains unknown whether TAOKs are involved in inflammatory disorders and, more broadly, if STE20-kinases are implicated with inflammaging. Among the three members of TAOs kinases, TAOK3 uniquely shows increased expression in the hematopoietic compartment, especially in macrophages [17, 18]. TAOK3 has already been found to be essential for various immune cell types and processes, such as T cell activity [19], marginal zone B cell formation [20], type 2 conventional dendritic cell homeostasis [21], and asthma [22]. Its function in macrophages and, more importantly, in chronic inflammation is unknown. Herein, we employ genetic and pharmacological tools to investigate the role of TAOK3 in the secretion of pro-inflammatory mediators. We found that aged knockout mice, especially females, had enlarged spleens. We associated splenomegaly with increased myeloid cell numbers and the commitment of hematopoietic progenitor cells toward myelopoiesis. Finally, the kinase activity of Taok3 limits the differentiation of macrophages and their polarization towards pro-inflammatory responses.

## Results

### Taok3 deficient mice develop severe skin ulcerations with age

**Fig. 1:**
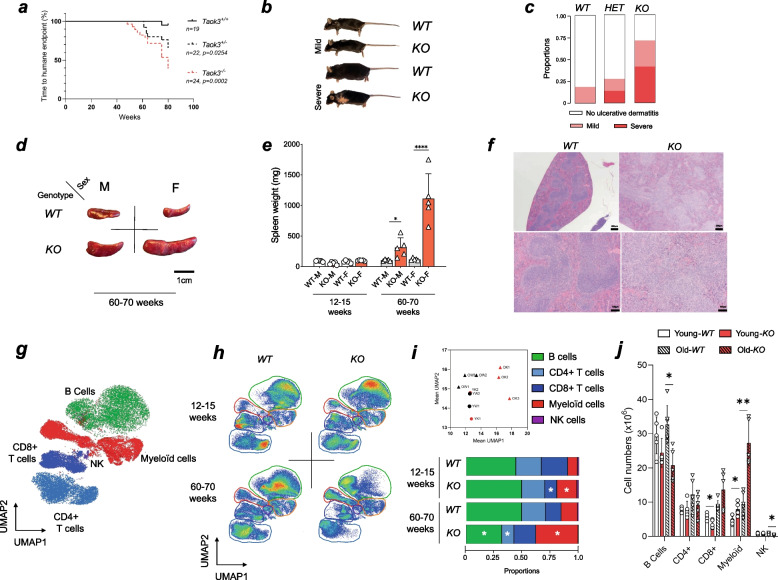
Taok3 deficient mice develop severe skin ulcerations and splenomegaly with age. **a-e** Longitudinal follow-up and macroscopic evaluations of Taok3^+/+^ (*WT*), Taok3^±^ (*HET*), and Taok3^−/−^ (*KO*) aged mice. **a** Curve reporting the time to humane endpoint between Taok3 genotypes. **b** Photographs of the skin ulcerations reported in the mice with the indicated genotype. **c** Bar chart representing the proportion of mice presenting severe, mild, or no skin ulcerations at the time of humane endpoint. **d** Photographs of spleens from 60–70-week-old male and female mice. Reference scale = 1 cm. **e** Bar plot representing the spleen weight (mg) of WT and KO animals stratified by sex and age group. **f** Micrograph of H&E-stained section from WT (left) and KO (right) spleens at 40X (top) and 100X (bottom) magnifications. **g** Color-coded UMAP clustering of splenic populations used to discriminate between B cell, Myeloid, NK, CD8 + T cell, and CD4 + T cell clusters. **h** Pseudo-color UMAP clustering of splenic populations from the indicated animals. **i** Downstream analyses of (top) dot plot representing the average UMAP 1 and 2 parameters from h and (bottom) bar plot representing the average proportions of the clustered populations within the global splenic population. **j** Bar plot of the total cell numbers of the populations described in i) (B cells, CD4 + T cells, CD8 + T cells, Myeloid cells and NK cells) in the spleen of young and old mice. **a-e, j** *n* = 10 animals per genotype. **f-i** Data representative of experiments realized on at least three independent litter pairs. Statistical analysis: **a** Log-rank test of simple survival analysis. **e** Two-way ANOVA with Dunnet’s multiple comparisons. **i-j** Multiple unpaired, bilateral T-tests

We studied the possible establishment of age-related inflammatory disorders using a cohort of *Taok3*^+*/*+^ (WT), *Taok3*^±^ (HET)*,* and *Taok3*^*−/−*^ (KO) littermates. Mice were followed-up until they reached the age of 80 weeks or 1.5 years. We first identified that knockout and heterozygous animals developed severe ulcerative dermatitis (UD), which necessitated euthanasia per-humane endpoint (Fig. 1a, b, c). The median survival for *Taok3*^+*/*+^, *Taok3*^±^*,* and *Taok3*^*−/−*^ animals was 79.5, 69.5, and 59 weeks respectively (Fig. 1a). In the vast majority of cases, UD lesions for both *Taok3*^±^ and *Taok3*^*−/−*^ animals was found around the neck and shoulder area, suggesting that lesions occur after scratching (Fig. 1b). Skin ulcerations are not uncommon in mice with C57/B6 background [23, 24].

### Splenic enlargement in Taok3 deficient mice is associated with increased myeloid cell frequency

Idiopathic UD is linked to behavioral, commensal, environmental, and inflammatory factors alike [24]. We graded the lesions based on previous reports [24] (Fig. 1c). We thus performed full-body necropsies on mice that did not have UD to pinpoint a plausible cause for the lesions further. Given the highly heterogeneous nature of the UD lesions within each group, we cannot rule out that other factors, such as aggressivity, stress and scratching intensity, are also plausible contributors to these lesions. All further analyses were also conducted in unaffected animals. We found that *Taok3*^*−/−*^ mice had enlarged spleens, which was more pronounced in female mice (Fig. 1d, e). We also observed loss of follicular architecture and cellular proliferation in the white pulp of the spleen (Fig. 1f). This meant that the splenomegaly was most likely driven by the accumulation of immune cells within the spleen. To investigate the immune populations responsible for this enlargement, we performed flow cytometric analyses on splenocytes of 12–15-week-old (Young) and 60–70-week-old (Old) non-lesioned animals. We used a 19-color flow cytometry panel to perform algorithmic population clustering using Uniform Manifold Approximation and Projection (UMAP) [25] (Fig. 1g). We found that both young and old Taok3 deficient mice had an increase in myeloid populations (Monocytes, neutrophils) (Fig. 1h, i). In older mice, this increase was also accompanied by a decrease in the frequency of lymphocyte populations (B cells, CD4 + T cells) (Fig. 1h, i). Differences between the genotypes were the most drastic in older mice, as demonstrated by the distance in the average UMAP1 parameter (Fig. 1i). Indeed, percentages of splenic myeloid cells were 9.3% and 19% in young wild-type and young knockout mice, respectively (Fig. 1i). On the other hand, it was 15% and 29% in older mice (Fig. 1i). These data suggest that Taok3 deficient mice accumulate myeloid cells in their spleens. This accumulation was found to be more drastic in older females.

### Enhanced age-related myeloid skewing in Taok3-deficient hematopoietic progenitor cells

**Fig. 2:**
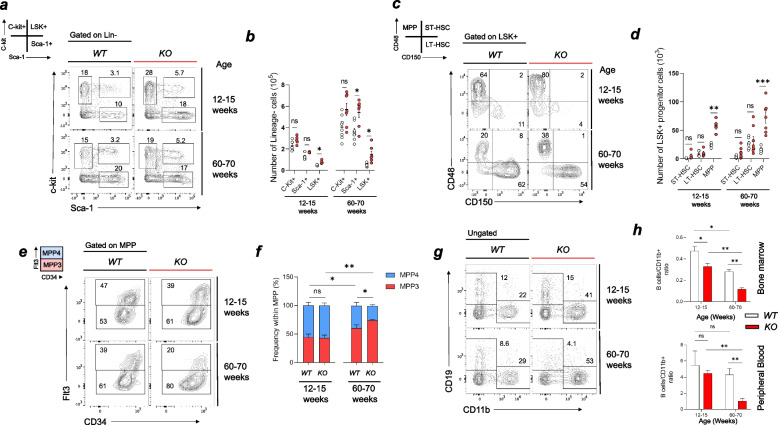
Shift in hematopoietic progenitor cell lineage commitment toward myeloid lineages in aged Taok3-/- mice. Flow cytometric analyses of mouse bone marrow hematopoietic stem cells. **a** Gating strategy to determine the C-Kit + , Sca-1 + and C-Kit + , Sca-1 + (LSK +) populations within lineage negative cells. Lineage was determined by the surface expression of one or more of these markers: CD3, TER119, CD11b, CD45R, GR-1. **b** Bar plot with individual values representing the total number of C-kit + , Sca-1 + , and LSK + cells. **c** Gating strategy to discriminate between MPP (CD48 + , CD150-), ST-HSCs (CD48 + , CD150 +), and LT-HSCs (CD48-, CD150 +) within the LSK + subpopulation **d)** Bar plot with individual values representing the total number of ST-HSCs, LT-HSCs, and MPPs. **e** Gating strategy used to differentiate between lymphoid-committed multipotent progenitors (MPP4) and myeloid-committed multipotent progenitors (MPP3) within the MPP compartment. **f** Bar chart representing the distribution between MPP4 and MPP3 in the global MPP population. **g** Gating strategy used to differentiate between B Cells and Myeloid cells in the bone marrow of young and old mice. **h** Bar plot with individual values representing the B cell (CD19 +)/ Myeloid cell(CD11b +) ratio in bone marrow (top) and peripheral blood (bottom). **a-g** Data representative of at least 5 independent litters (*n* = 5/genotype/age-group). h) Young groups have *n* = 6 (WT) and *n* = 5 (KO) whilst old have *n* = 3 (WT) and *n* = 4(KO). Chart error bars represent mean ± SEM. Statistical analysis: **b**, **d**, **f**, **h** Multiple unpaired, bilateral T-tests. ns, non-significant. *p ≤ 0.05, **p ≤ 0.01, ***p ≤ 0.001, ****p ≤ 0.0001

Since we observed a decrease in lymphoid cells in favor of myeloid populations in the spleens, we wondered whether Taok3 deletion affected myelopoiesis in the bone marrow. To investigate this, we employed flow cytometric analysis of hematopoietic progenitor cells in the bone marrow of young and old mice. We found that the frequency and numbers of progenitor LSK + (Lineage-, Sca-1 + , C-kit +) cells were increased in both young and old knockout animals (Fig. 2a, b). We next measured the proportions of long-term HSCs (LT-HSCs; LSK + CD150 + CD48 +), short-term HSCs (ST-HSCs; LSK + CD150 + CD48 +), and multipotent progenitors (MPPs; LSK + CD150-CD48 +). In both age groups, a significant increase in the frequency and numbers of MPPs was observed in KO versus WT animals (Number of MPPs (× 10^3^): Young-WT: 24.4; Young-KO 58 (1-fold increase), Old-WT: 15.5; Old-KO: 72.3 (4.8-fold)) (Fig. 2c, d). It is well known that aging favors myeloid lineage differentiation rather than lymphoid lineage commitment in hematopoietic stem cells (HSCs) [26, 27]. This skewing can also be observed within MPP progenitors. Indeed, MPP3 progenitors are polarized toward myeloid lineages, whereas MPP4s are polarized toward lymphoid lineages [28, 29]. We found no differences between the proportions of MPP3 and MPP4 cells in young mice (Fig. 2e, f). As expected, there was an increase in MPP3 frequency in aged mice. This increase was more dramatic in knockout animals, which implies further commitment towards myelopoiesis rather than lymphopoiesis (Fig. 2f). The B-cell to myeloid (CD11b +) ratio was found to be decreased in the bone marrow and peripheral blood of Taok3 deficient mice (Fig. 2h). The skewing towards myelopoiesis could at least partly explain why these mice had more myeloid cells in their spleens, bone marrow, and peripheral blood. Altogether, those results indicate that Taok3 limits age-related myelopoiesis commitment.

### Taok3 deletion increases pro-inflammatory cytokine levels in the blood

**Fig. 3:**
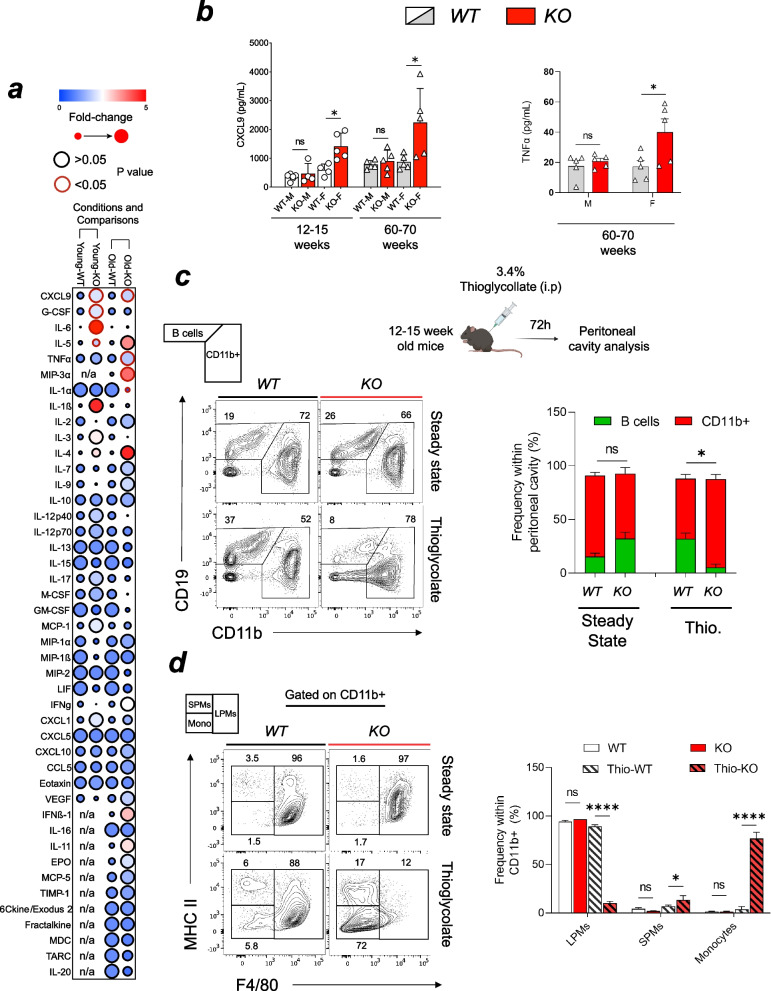
Sex-dependant increase in pro-inflammatory and chemotactic factor in Taok3 deficient mice. **a** Serum cytokine and chemokine measurements in young and aged mice reported by fold change (KO/WT within the same age group) and associated *p*-value. **b** Bar chart illustrating CXCL9 (left) and TNFα (right) levels, stratified by the sex, age, and genotypes of the animals. **c** (from top left to bottom right) Quantifying monocytic migration potential in a model of acute peritonitis in vivo. The gating strategy was used to define the major B cell (CD19 + , CD11b-) and CD11b + populations found in the peritoneal cavity in steady state and during Thioglycollate (Thio.) induced peritonitis. Bar chart representing the distributions of B cells and CD11b + cells within the peritoneal cavity. These experiments were conducted in female mice. **d** Gating strategy to characterize resident large peritoneal macrophages (LPMs) from recruited small peritoneal macrophages (SPMs) and recruited monocytes (mono) within the CD11 + population. Associated bar chart representing the frequency of each subpopulation. **a-b** Data representative of *n* = 5 animals/genotype/sex/age-group. **c-d** Data representative of independent experiments conducted on a total of *n* = 6 mice/condition/genotype. Chart error bars represent mean ± SEM. Statistical analysis: **a-d** Multiple unpaired, bilateral T-tests. ns, non-significant. *p ≤ 0.05, **p ≤ 0.01, ***p ≤ 0.001, ****p ≤ 0.0001

It remained unknown, however, whether the function of those myeloid cells was increased and how they could negatively impact survival and inflammation. We measured the levels of pro-inflammatory factors in 12–15 weeks old (Young) and 60–70 old (Old) mice. To do so, we performed cytokine multiplexing in the serum of those animals at a steady state (Fig. 3a). Out of the 44 markers tested, we found that CXCL9 (2.1-fold), G-CSF (2.1-fold), IL-6 (12.9-fold) and IL-5 (1.87-fold) levels were elevated in young knockout versus young wild-type animals (Fig. 3a). We also detected a trend towards increased levels of IL-1β in young KO animals (although not statistically significant). The increase of IL-1β and IL-6 is consistent with a systemic inflammatory trigger preceding the phenotypes described earlier (UD and splenomegaly). In older mice, we found elevated CXCL9 (1.9-fold), TNFα (1.7-fold), and MIP-3β (3.93-fold). There was also a trend in both young and old KOs towards increased IL-4 levels, which goes in line with that UD lesions are a result of increased Type I hypersensitivity. Conversely, we found decreased levels of IL-1α (-70%) in older Taok3^−/−^ mice (Fig. 3a). We next stratified the levels of those cytokines/chemokines by sex to determine whether those factors could be implicated in the sex-dependent observation shown before. As expected, only female knockout mice had a significant increase in their CXCL9 and TNFα levels compared to female wild-type (Fig. 3b). We did not detect significant sex-dependent associations in the other differentially secreted factors (IL-6, IL-5, MIP-3β, and IL-1α). These observations indicate that Taok3 negatively modulates the production of pro-inflammatory cytokines and that sex-dependent factors modulate this function. These pieces of evidence point to Taok3^−/−^ mice having an accentuation of normal-age-related inflammation, rather than specific pathologies.

### Increased monocyte trafficking in Taok3−/− mice with acute peritonitis

In addition to its role in myeloid cell differentiation and function, CXCL9 is a major factor in the leukocyte trafficking and migration [30, 31]. We, therefore, investigated whether Taok3 deficient mice had increased capacity for myeloid cell transmigration in vivo. To do so, we induced acute peritonitis with intraperitoneal (i.p) injections of 3.4% thioglycollate. Under steady-state conditions, the peritoneal cavity is mainly populated by resident large peritoneal macrophages (LPMs) (CD11b + , F4/80 +) and B cells. Inflammation induces the transmigration of monocytes (CD11b + , F4/80^lo^, MHC II +) to the peritoneal cavity. These monocytes then continuously differentiate into small-peritoneal macrophages (SPMs) (CD11b + , F4/80^lo^, MHC II +), which secrete pro-inflammatory mediators, such as TNFα [32]. We did not observe any differences in the proportion of macrophages and B cells under steady-state conditions (Fig. 3c). However, the induction of acute peritonitis in Taok3 deficient mice significantly increased the proportion of CD11b + cells compared to wild type (Fig. 3c). Further analysis revealed that the vast majority of cells (89%) were not resident LPMs, but either SPMs (17%) or monocytes (72%) (Fig. 3d). For comparison, wild-type animals had 6% of SPMs, 5.8% of monocytes and 88% of resident LPMs in their peritoneal cavity (Fig. 3d). It implies that most of the cells that populated the peritoneal cavity of Taok3^−/−^ mice after the induction of peritonitis migrated from the periphery. This result suggests that under normal circumstances, Taok3 limits the recruitment of monocytes to sites of inflammation.

### Taok3 negatively modulates the differentiation of bone marrow-derived macrophages and their secretion of pro-inflammatory cytokines

**Fig. 4:**
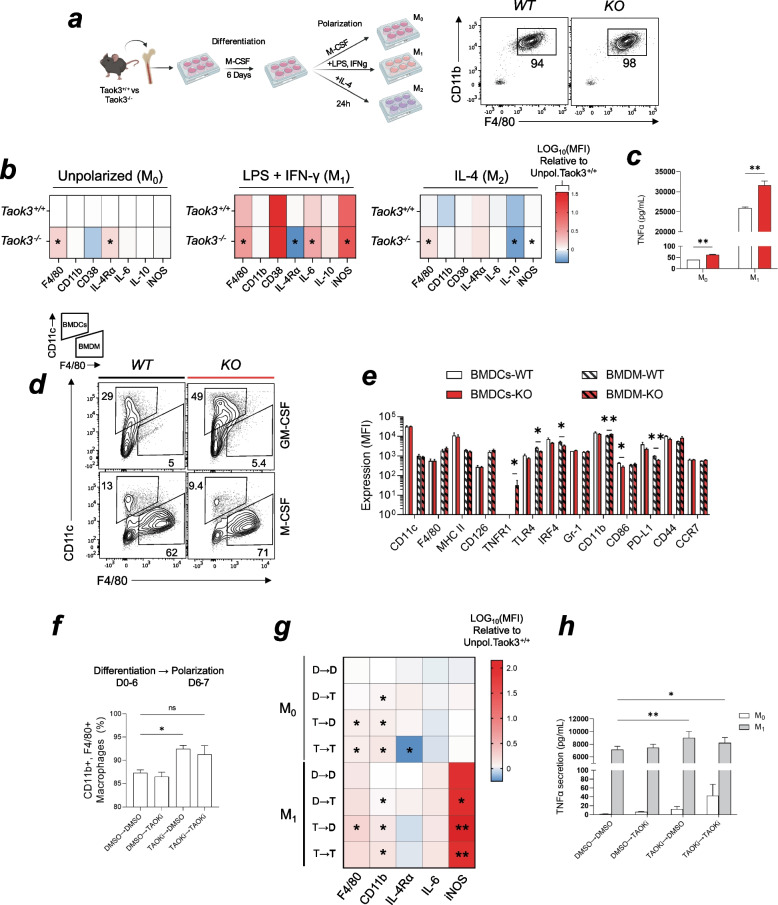
Taok3 deletion or inhibition increases differentiation of macrophages and their production of pro-inflammatory response factors. **a** Schematic representation of the method employed to differentiate and polarize bone marrow derive- macrophages (BMDMs). Representative flow cytometry plot showing the purity of BMDM cultures, as assessed by the frequency of CD11 + , F4/80 + macrophages. **b** Heatmaps representing the LOG_10_ (Fold change) relative to Taok3.^+/+^ unpolarized M_0_ using the mean fluorescence intensity by flow cytometry. Statistical comparisons are made between genotypes between the same polarization regimens. **c** Quantification of the TNFα levels in BDMSs from b) using ELISA. **d** Gating strategy used to discern between GM-SCF grown BMDCs (CD11c +) and M-CSF differentiated BMDMs (F4/80 +). **e** Flow cytometric analysis of the mean expression (mean fluorescence intensity) of surface molecules and transcription factors in BMDCs and BMDMs. **f** Bar plot of the frequency of differentiated macrophages, depending on the phase at which the cells were treated with the inhibitor. Differentiation (Day 0–6) → Polarization (Day 6–7). **g** Heatmaps representing the LOG_10_ (Fold change) relative to DMSO → DMSO unpolarized M_0_ using the mean fluorescence intensity by flow cytometry. Statistical comparisons are made between genotypes between the same polarization regimens against the respective DMSO → DMSO group. **h** Quantification of the TNFα levels in BDMSs from **g**) using ELISA. Data representative of at least three independent experiments. Chart error bars represent mean ± SEM***.*** Statistical analysis: **a-h** Multiple unpaired, bilateral T-tests. ns, non-significant. *p ≤ 0.05, **p ≤ 0.01, ***p ≤ 0.001, ****p ≤ 0.0001

We next wondered whether Taok3 deficiency directly affected macrophages' function and pro-inflammatory cytokine production. We used M-CSF at 30 ng/mL for 6 days to differentiate (CD11b + , F4/80 +) macrophages from bone marrow cells (Fig. 4a). To unravel the role of this kinase in macrophage function, we then polarized those bone marrow-derived macrophages (BMDMs) to unpolarized M_0_, polarized M_1_, and M_2_ macrophages. Activation of macrophages with LPS and IFN-γ (100 ng/mL and 20 ng/mL respectively for 24 h) yields “classically” activated or M_1_ macrophages that produce pro-inflammatory cytokines such as IL-6 and TNFα [33]. Conversely, adding IL-4 (20 ng/mL for 24 h) will promote the polarization of "alternative" or M_2_ macrophages that have essential anti-inflammatory functions [33]. M_0_ macrophages correspond to unpolarized BMDMs that received M-CSF instead of the aforementioned polarization cocktails. We observed that Taok3-deficient M_1_ polarized BMDMs had increased expression of F4/80, IL-6, and inducible nitric oxide synthase (iNOS) (Fig. 4b). These cells also had decreased expression of IL-4 receptor alpha chain (IL-4Rα), a molecule responsible for mediating anti-inflammatory signals. These results, combined with the fact that M_2_ polarized macrophages had lower IL-10 and higher iNOS expression, indicate that Taok3 deficiency favors pro-inflammatory rather than anti-inflammatory responses in polarized macrophages originating from bone-marrow progenitors. M_0_ and M_1_ macrophages had increased TNFα production, as measured in the supernatants of those cultures by ELISA (Fig. 4c). We also observed significant changes in the expression of F4/80 in M_0_ BMDMs, which might imply that Taok3 alters not only the polarization but also the differentiation of macrophages from progenitor cells. We next wondered whether the absence of Taok3 could modulate the differentiation of other myeloid cell types, such as dendritic cells. We used GM-CSF at 20 ng/mL to induce the differentiation of bone marrow-derived dendritic cells (BMDCs) over a 6-day period. These BMDCs are characterized by high levels of CD11c at their surface. The purpose of this experiment was to measure whether Taok3 deletion also impacted Dendritic cell differentiation. In these experiments, we noticed an increase in the frequency of both BMDCs (GM-CSF) and BMDMs (M-CSF) populations after six days of differentiation (Fig. 4d). It is therefore possible that under normal conditions, Taok3 limits the effects of GM-CSF and M-CSF in myeloid cell differentiation. To further characterize these bone marrow-derived cells, we quantified the expression of receptors and transcription factors. In BMDCs, only the CD86 co-receptor was found to be significantly decreased in KO vs. WT cells (Fig. 4e). BMDMs, on their part, had reduced levels of TLR-4, the pattern recognition receptor that binds LPS, interferon response factor 4 (IRF4) and PD-L1. Conversely, Knockout BMDMs had higher levels of TNFR1 and CD11b (Fig. 4e). The decrease in IRF4 aligns with the hypothesis that Taok3 deficiency limits alternative polarization response in macrophages, given that IRF4 is crucial for shaping the phenotype of M_2_ macrophages [34].

Given that bone marrow progenitor cells were shown to be skewed towards myelopoiesis, it remained possible that alternative transcriptional programs were already found in precursors of BMDMs before differentiation with M-CSF. We thus used a small molecule inhibitor of TAO- family kinases, compound-43 [35], to validate our previous findings and to make two demonstrations: 1. That the catalytic/kinase activity of Taok3 is important in controlling TNFα production and M_1_ pro-inflammatory responses and 2. that those changes are intrinsic to differentiating macrophages and not a result of differential gene expression in progenitors. Using 5 µM of compound-43, we tested whether the phenotype relied on inhibition during the differentiation phase (Day 0–6) or the polarization phase (Day 6–7). We used four different treatment conditions: DMSO → DMSO (D → D), DMSO → TAOKi (D → T), TAOKi → DMSO (T → D), and TAOKi → TAOKi (T → T) that represent the phase at which the drug/vehicle was added (Differentiation → Polarization). As expected, only the BMDMs treated during their differentiation phase (TAOKi → DMSO) had an increased frequency of BMDMs compared to the DMSO → DMSO control group (Fig. 4f). We next polarized those BMDMs to M_1_ macrophages using the same method as described previously to validate previous findings in a macrophage-intrinsic model. We observed that administration of TAOK inhibitor, regardless of condition, consistently increased CD11b expression (Fig. 4g). This increase was also accompanied by an increase in F4/80 in TAOKi → DMSO M_0_ and M_1_ macrophages.

Most importantly, inhibition of Taoks resulted in a significant increase in iNOS expression, which was more pronounced in T → D and T → T conditions (Fig. 4g). iNOS is a critical enzyme in the macrophage inflammatory response and is responsible for inducing bacterial killing via nitric oxide (NO) synthesis [33, 36]. We also quantified TNFα levels in the supernatants of those conditions using ELISA. We found that only M_1_ macrophages that had received TAOKi during their differentiation phase (T → D and T → T) had increased concentrations of TNFα (Fig. 4h). This result not only validates the previous findings in a cell-intrinsic manner but also provides evidence for the importance of the kinase activity of Taok3 during the differentiation of macrophages. Altogether, these findings demonstrate that Taok3 negatively modulates the differentiation of bone marrow-derived macrophages and their secretion of pro-inflammatory cytokines IL-6 and TNFα. In addition to this function, in vitro and in vivo results suggest that Taok3 has a role in limiting sex and age-related inflammation through its role in leukocytes.

## Discussion

With the increase in life expectancy worldwide, the number of aged individuals is set to rise to historic highs in the following decades. With increasing age comes inflammaging, an incompletely understood condition that increases the risk of chronic diseases. Here, we uncovered that an unexpected gene, TAOK3, plays a role in limiting inflammaging by negatively modulating the polarization of macrophages towards pro-inflammatory responses. We identified that the kinase activity of the enzyme is necessary for limiting macrophage differentiation in-vitro. Our study supports the mounting evidence for the implication of innate immune cells and their receptors in chronic inflammation [37]. Other essential macrophage functions we did not cover include efferocytosis and debris clearance within the organism. The importance of those functions with inflammaging has already been demonstrated [2, 38]. Our findings support that genetic factors can induce susceptibility to pro-inflammatory cytokine and chemokine production in a sex-dependent manner. Such associations are important for understanding other diseases, such as rheumatoid arthritis (RA) and systemic lupus erythematosus (SLE), given that those diseases disproportionately affect women. These diseases, along with inflammatory skin conditions, strongly implicate the pro-inflammatory functions of TNF-α [39]. Our findings involving TAOK3, TNF-α, and ulcerative dermatitis in mice, further support this implication. TAOK3 single nucleotide polymorphisms (SNPs) have also been linked with an increased risk of SLE [40]. The rs428073 SNP encodes a C-T missense variant causing the S47N substitution. The functional role of this mutation remains unknown, but the variant is near the predicted ATP binding site. Thus, it may interfere with the catalytic activity of the kinase. Our study demonstrates that the Ser/Thr kinase activity of TAOK3 is important in limiting pro-inflammatory responses in macrophages. Several groups have shown that inhibiting the enzyme induces anti-cancer responses [35, 41, 42]. A demonstrated relationship between NF-κB mediated signaling and TAOK3 could also imply the presence of inflammatory responses in cancer cells [43]. TAOK3 has also been implicated in skeletal mineralization by modulating the differentiation of osteoblasts [44]. This brings essential implications, given the established link between age-related inflammation and bone disorders, such as osteoporosis [45]. TAOK3, and possibly other STE-20 kinases, finds itself at the center of an association between inflammation, sex-specific associations, and innate immunity. We are limited in our interpretation of our data, given that we do not possess a tissue-specific knockout model. Such a model would be paramount in dissecting other macrophage-specific functions, such as efferocytosis and phagocytosis. We are also limited in the statistical power of our study. We also uncovered an unexpected role for TAOK3 in hematopoiesis. It remains unclear, however, at which precise stage does Taok3 participates in hematopoiesis. The cell-autonomous effects of the kinase in this setting also remain unknown. Many different molecular mechanisms could explain the phenotypes observed here. TAOK3 has already been linked to Notch signaling in other immune cell types [20, 21]. However, it seems unlikely that this mode of action is central here, given the reported positive role of TAOK3 in Notch signaling and the importance of Notch signaling in the M_1_ polarization [46, 47]. Overall, our findings align with previous reports stating that other STE-20 enzymes negatively modulate macrophage function and pro-inflammatory responses [16, 48, 49]. Thus, like the other STE-20 kinases, TAOK3 could be involved in innate receptor signaling, such as TLR receptors, through interaction with TRAF proteins [16]. TAOK3 has been previously demonstrated to interact with TRAF2, although it was not in the context of TLR signaling [50]. TAOK1, which is 73.56% identical in amino acid sequence to TAOK3, was shown to interact with TRAF6 during IL-17-mediated inflammation [48]. These reports, combined with recent evidence showing that some TLRs are involved in sex-dependent inflammatory and auto-immune disorders, provide further directions for studying the mechanism by which TAO kinases operate [37, 51, 52].

## Methods

### Mice

Whole-body Taok3 KO mice were purchased from Jackson Laboratories MMRRC stock #43,790. These mice were generated by the KOMP team using a CRISPR-generated KO mutant of the Taok3 kinase by deleting exon 6, leading to a frameshift mutation causing an early stop codon [53]. This frameshift nullifies protein expression but not mRNA transcription. Taok3^−/−^ mice were bred with background-compatible C57/Bl6 mating partners. Control animals for *Taok3*^*−/−*^ mice were *Taok3*^+*/*+^ littermates from the heterozygous breedings mentioned above. Animals were housed and bred in specific-pathogen-free facilities at the Comparative Medicine and Animal Resources Centre (CMARC) at McGill University in accordance with the Canadian Council on Animal Care Guidelines.

### Histology

Tissues were fixed in 10% Buffered formalin. From fixed tissue, representative sections were trimmed and processed for paraffin embedding. Paraffin blocks are cut at a width of approximately 4 µm onto glass slides and stained with hematoxylin and eosin.

### Flow cytometry

Staining and washing were performed in round bottom polystyrene 5 mL tubes (Falcon, 14–959-6). Unless otherwise stated, intracellular staining was performed with the BD biosciences Fixation/Permeabilization solution (BD Biosciences, 554,714) according to the manufacturer’s protocol. Beferdin A (BD Biosciences, 555,029) solution was used as the protein transport inhibitor during intracellular stainings. A 1:1000 dilution was incubated directly in cell culture plates for 4 h before stainings. Data were acquired using the Cytek Aurora (19-color panel) and the BD 5-laser LSR FORTESSA. Data were analyzed using FlowJo. V10 software.

Antibodies: Bone marrow HSCs: Anti-CD3-Biotin(Biolegend,1:400),TER119-Biotin(Biolegend,1:400),CD11b-Biotin (Biolegend,1:400), CD45R-Biotin(Biolegend,1:400), GR-1-Biotin (Biolegend,1:400), C-kit-APC (Biolegend,1:400), Sca-1-PECY7 (Biolegend,1:400), CD48-BV605 (Biolegend,1:300), CD150-BV450 (Biolegend,1:300), FLT3-PE (Biolegend,1:300), CD34-FITC (Biolegend,1:300), D16/32-APCCY7 (Biolegend, 1:300), IL7RA-BV785 (Biolegend,1:300). Spleens: CD19-AF647 (Biolegend, 1:1600), B220-AF700 (Biolegend, 1:800), IgM -BV605 (Biolegend, 1:200), IgD-PerCPCy5.5 (Biolegend, 1:400), CD3-APC/Fire810 (Biolegend, 1:150), CD4-PE/Dazzle 594 (Biolegend, 1:800), CD8-Pacific Blue (Biolegend, 1:600), CD44-BV421 (Biolegend, 1:400), CD62L-BV786 (BD, 1:1200), NK1.1-FITC(Biolegend, 1:300), TCRb-APC (Biolegend, 1:200), Ter119-BV650(Biolegend, 1:400), CD71-PE(Biolegend, 1:800), Ly6G-PE/Cy7 (Biolegend, 1:400), Ly6C-APC/Cy7(Biolegend, 1:400), CD11b-SparkYG593 (Biolegend, 1:800), CD11c-BV510(Biolegend, 1:150), F4/80-PE/Cy5 (Biolegend, 1:600), MHCII Spark Blue 550(Biolegend, 1:800), F4/80-PECY5 (Biolegend, 1:600), CD11c -PE (Biolegend, 1:600), GR1 (bv650) biotin (Biolegend, 1:500), CD126-PECY7(Biolegend, 1:400), CD86 (BV786)(Biolegend, 1:300), PD-L1-APC(Biolegend,1:1:200),CD44-PECY7 (Biolegend, 1:1:300),MHC1-PERCPCY5.5(Biolegend,1:1:200), CCR7-BV421 (Biolegend, 1:1:300),CD11b-FITC (Biolegend, 1:500), IL4-Rα-PECY7 (Biolegend, 1:300),IL-6-PE(Biolegend, 1:100), IRF4-BV421 (Biolegend, 1:100), iNOS-APC (Biolegend, 1:300).

### Generation of BMDMs and BMDCs

Bone marrow from both femurs and tibiae was harvested in Dulbecco's High Glucose Modified Eagles Medium (DMEM) (Cytiva, SH30022FS) supplemented with 10% heat-inactivated FBS (Life technologies, 12,483,020). Marrows were filtered through a 70 μm Nylon cell strainer (Corning, 07–201-431) to remove solid fragments. The filtrate was centrifuged at 300 × g for 5 min at 4 °C. Cells were subsequently seeded in 2 ml DMEM supplemented with 10% hi-FBS, 1% non-essential amino acids (Gibco, 11,140,050), 1 mM sodium pyruvate (Gibco, 11,360,070), 2 mM L-Glutamine (Glutamax) (Gibco, 35,050,061), 100U/ml penicillin–streptomycin (Gibco, 15,140,122) in 6-well plates at a density of 1 × 10^5^ cells/mL. Recombinant murine M-CSF (Preprotech, 315–02) was added directly to the wells on days 0, 3, and 6 at a final concentration of 30 ng/mL. The same method was employed to generate BMDCs, but with 20 ng/mL of recombinant murine GM-CSF (Preprotech, 315–03) and 5 ng/mL of recombinant murine IL-4 (Preprotech, 214–14). BMDCs were in their immature “unactivated” state during that study. Cells were harvested by gentle scraping and washing with PBS. Washed cells were then used for downstream applications.

For the polarization, 100 ng/mL of LPS (LPS from E. coli O111:B4, InVivogen, tlrl-3pelps) and 20 ng/mL of recombinant murine IFNγ (Preprotech, 315–05) were used to induce M_1_ macrophages for 24 h. For M_2_ macrophages, 20 ng/mL of recombinant murine IL-4 (Preprotech, 214–14) was added to the culture wells for 24 h.

The TAO kinases inhibitor, compound 43, was diluted in DMSO (water insoluble) and purchased from Cayman chemicals (Ref: 25632).

### Enzyme-linked immunosorbent assays (ELISA) and cytokine/chemokine multiplexing

Supernatant from BMDM cultures was collected on day 7 ( 24 h post-polarization) and kept at -80C until downstream analysis. Mouse IL-6 (Biolegend, 431,301) and TNFα (Biolegend, 430,904) were used as per the manufacturer's protocol.

For cytokine multiplexing, serum was collected through cardiac puncture using 1 mL syringes and 25G × 1 needles (BD biosciences). Blood was transferred to S-Monovette® Serum Gel, 1.1 mL collection vessels (Sarstedt, 06.1667.001) and was left to cloth for 30 min at room temperature. Vessels were then centrifuged at 2000 × *g* for 10 min. Supernatants (serum) were collected and stored in new tubes at -80C until downstream analysis. For cytokine multiplexing, serum was diluted onefold before being sent to EVE Technologies, Calgary, Alberta, Canada, for cytokine multiplexing with a Bio-Plex 200.

### Statistical analysis

GraphPad Prism software v9.0a (GraphPad Software) was used to prepare graphs and perform statistical analysis. We used a two-tailed student T-test for pairwise comparisons to determine statistical differences between distributions. ns, not significant, *p ≤ 0.05, **p ≤ 0.01, ***p ≤ 0.001, ****p ≤ 0.000.

## Supplementary Information

**Additional file 1: Sup. Fig. 1.** Additional data pertaining to Fig. 1. a) Dot plot indicating the mean weight of male and female mice in grams (g) in different age groups (young, adult, old). b) Bar chart indicating the mean age at humane endpoint between male and female Taok3^-/-^ mice (top). Bar chart indicating the proportion of each sex within the dead animal group. c) Pathology report for brain, lung, liver, spleen, lymph node, and colon tissues fixed in formalin and analyzed with H&E staining. d) Micrograph of H&E-stained section from WT (left) and KO (right) axillary lymph nodes at 40X (top) and 100X (bottom) magnifications. e) n/nx plots for the parameters used to calculate the UMAP in Fig. 1g. Right side table represents the representative markers used to define major splenic populations. Data representative of at least three independent litters. Each animal represents one data point (a-b). Statistical analysis: a) Two-way ANOVA with Dunnet’s multiple comparisons. (b) unpaired, bilateral T-tests. Chart error bars represent mean±SEM. Non-significant differences, ns. *p≤0.05, **p≤0.01, ***p≤0.001, ****p≤0.0001.

**Additional file 2: Sup. Fig. 2.** Flow cytometric analysis of Neutrophil and monocyte subsets in peripheral blood. Gating strategy employed to discriminate between neutrophils (CD11b+, SSC-A^hi^, Ly6C^+^) and monocytes (neutrophils (CD11b+, SSC-Alo, Ly6C+). Bar chart representing the frequency of neutrophils and monocytes within the CD11b+ population. Statistical analysis: Multiple unpaired, bilateral T-tests.

**Additional file 3: Sup. Fig. 3.** Additional cytokine profiling in Taok3^+/+^ and Taok3^-/-^ mice. a) Cytokine multiplexing quantification of TARC/CCL17 and MDC/CCL22 levels in serum of mice stratified by genotype and sex. b) IL-6 mean fluorescence expression in F4/80+, CD11b+ LPMs in steady state mice. Each animal represents one data point. Bar chart represents mean +/- SEM. Statistical analysis: (a-b) Multiple unpaired, bilateral T-tests.

## Data Availability

The datasets used and/or analyzed during the current study are available from the corresponding author on reasonable request.
