# Supplementary figures and images for: TAOK3 limits age-associated inflammation by negatively modulating macrophage differentiation and their production of TNFα
Source: Immun Ageing. 2023 Jul 3;20:31. doi: 10.1186/s12979-023-00350-y (PMC10316641; doi:10.1186/s12979-023-00350-y)

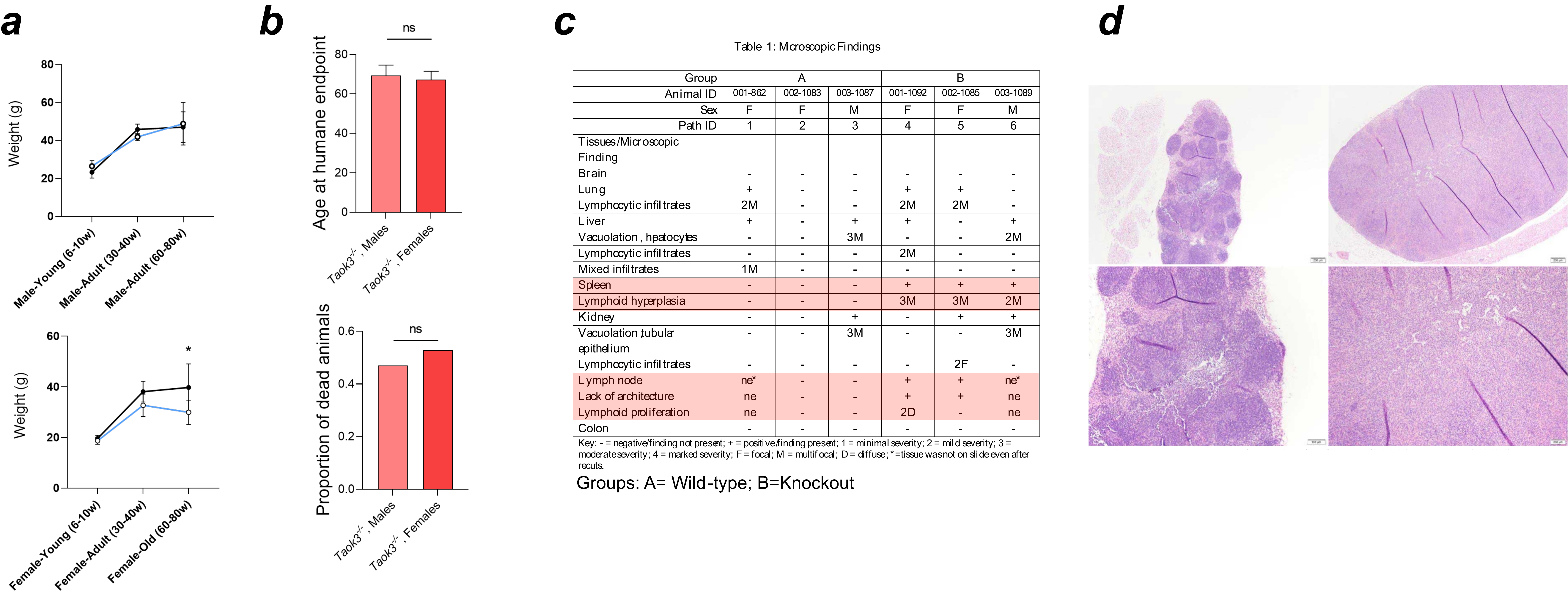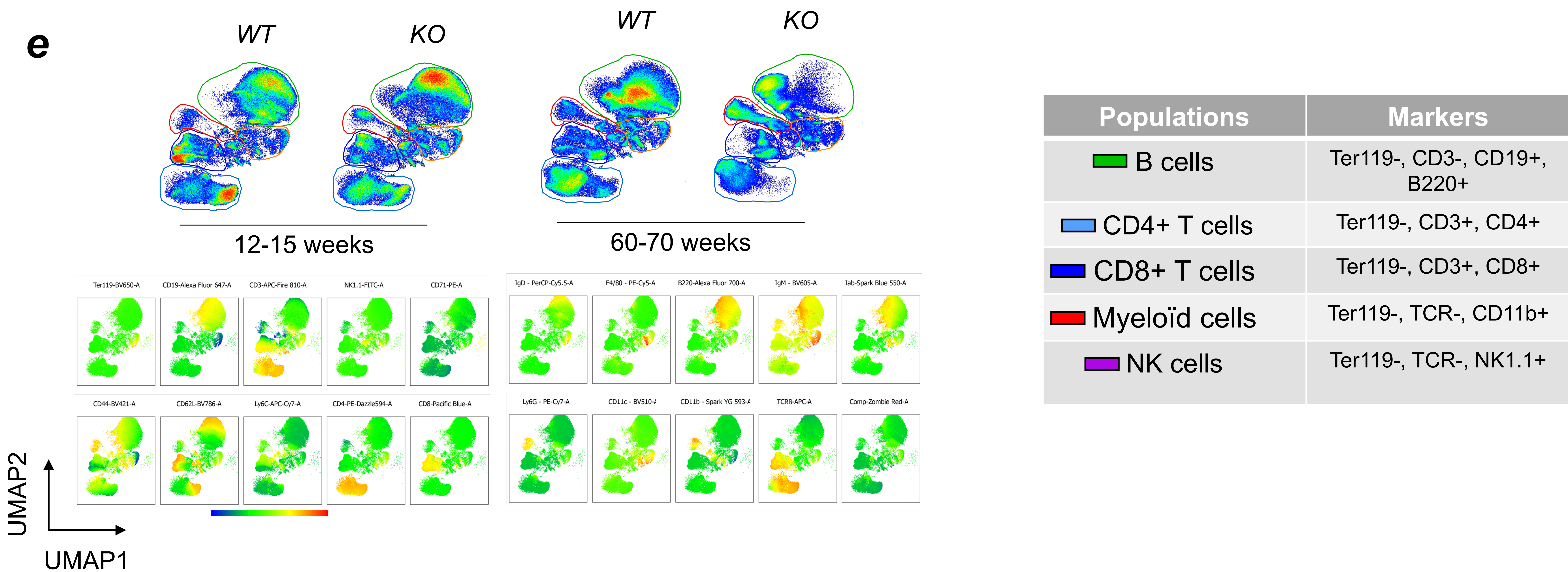

Supplement: Supplementary file 1 — Additional file 1: Sup. Fig. 1. Additional data pertaining to Fig. 1. a) Dot plot indicating the mean weight of male and female mice in grams (g) in different age groups (young, adult, old). b) Bar chart indicating the mean age at humane endpoint between male and female Taok3-/- mice (top). Bar chart indicating the proportion of each sex within the dead animal group. c) Pathology report for brain, lung, liver, spleen, lymph node, and colon tissues fixed in formalin and analyzed with H&E staining. d) Micrograph of H&E-stained section from WT (left) and KO (right) axillary lymph nodes at 40X (top) and 100X (bottom) magnifications. e) n/nx plots for the parameters used to calculate the UMAP in Fig. 1g. Right side table represents the representative markers used to define major splenic populations. Data representative of at least three independent litters. Each animal represents one data point (a-b). Statistical analysis: a) Two-way ANOVA with Dunnet’s multiple comparisons. (b) unpaired, bilateral T-tests. Chart error bars represent mean±SEM. Non-significant differences, ns. *p≤0.05, **p≤0.01, ***p≤0.001, ****p≤0.0001. [file 12979_2023_350_MOESM1_ESM.pdf]

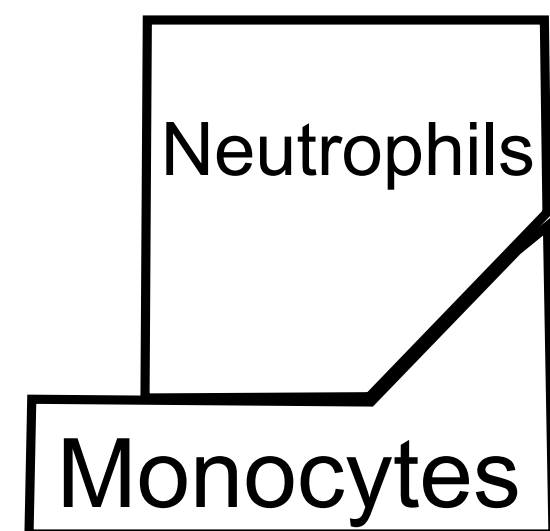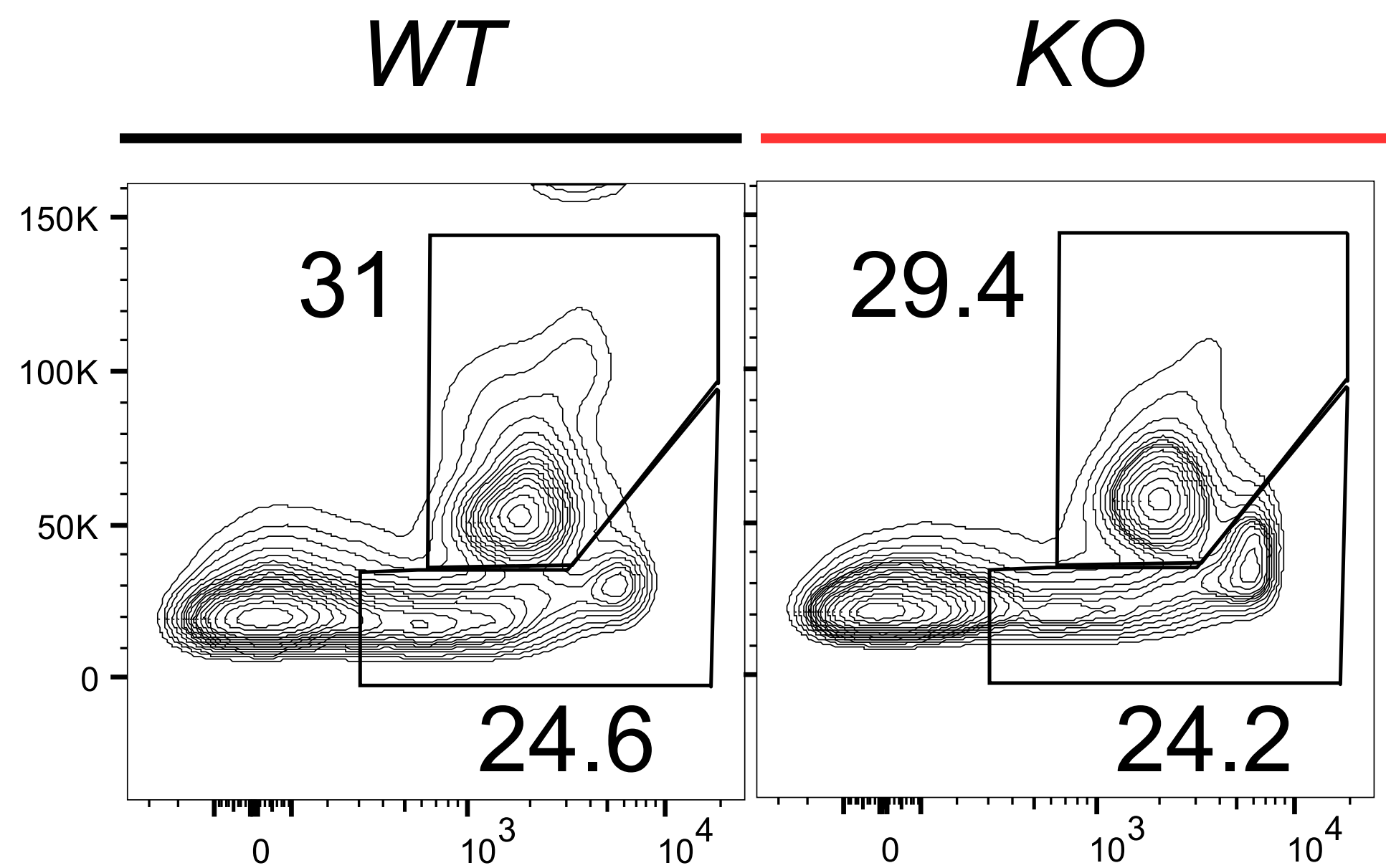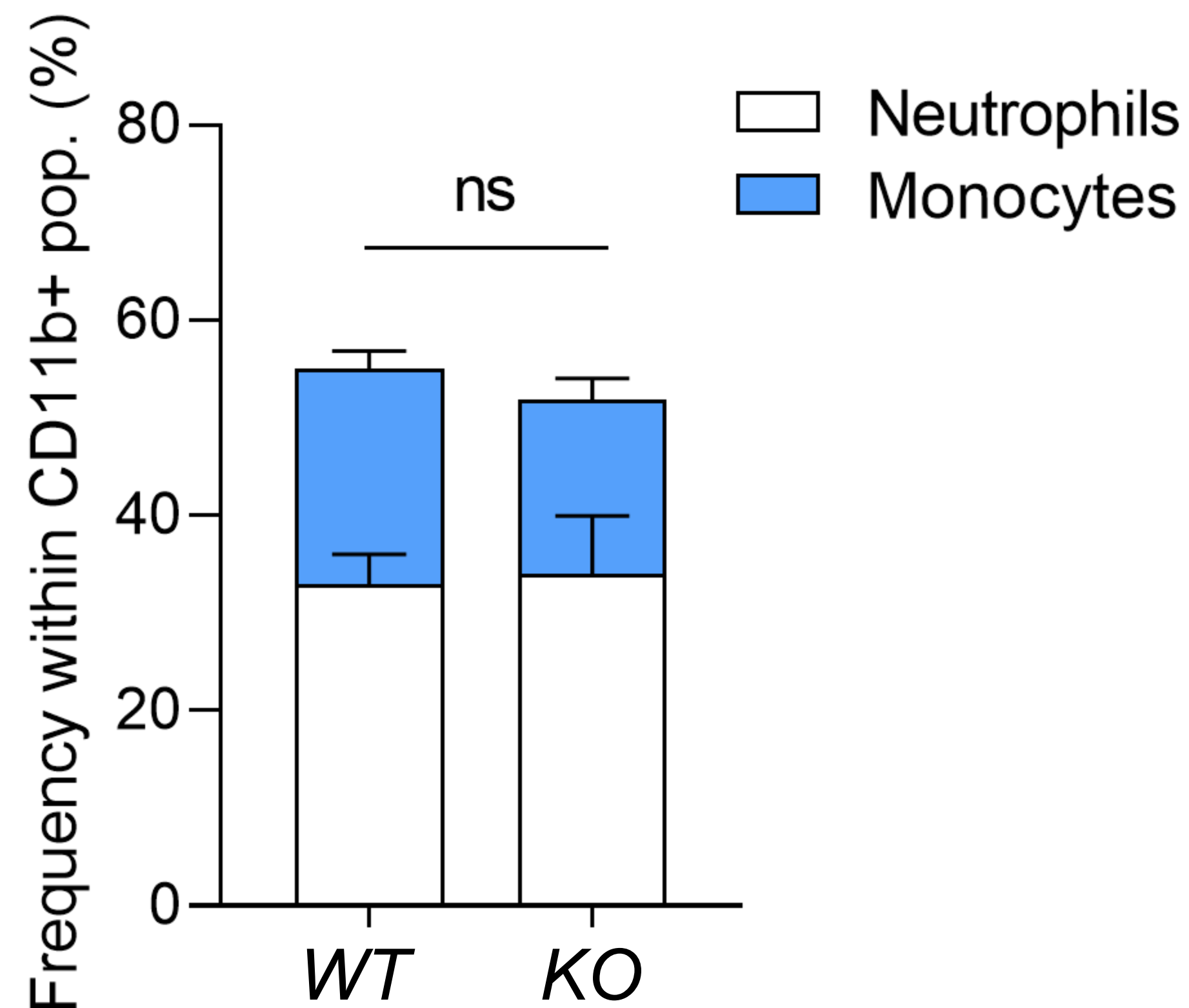

Peripheral blood

Supplement: Supplementary file 2 — Additional file 2: Sup. Fig. 2. Flow cytometric analysis of Neutrophil and monocyte subsets in peripheral blood. Gating strategy employed to discriminate between neutrophils (CD11b+, SSC-Ahi, Ly6C+) and monocytes (neutrophils (CD11b+, SSC-Alo, Ly6C+). Bar chart representing the frequency of neutrophils and monocytes within the CD11b+ population. Statistical analysis: Multiple unpaired, bilateral T-tests. [file 12979_2023_350_MOESM2_ESM.pdf]

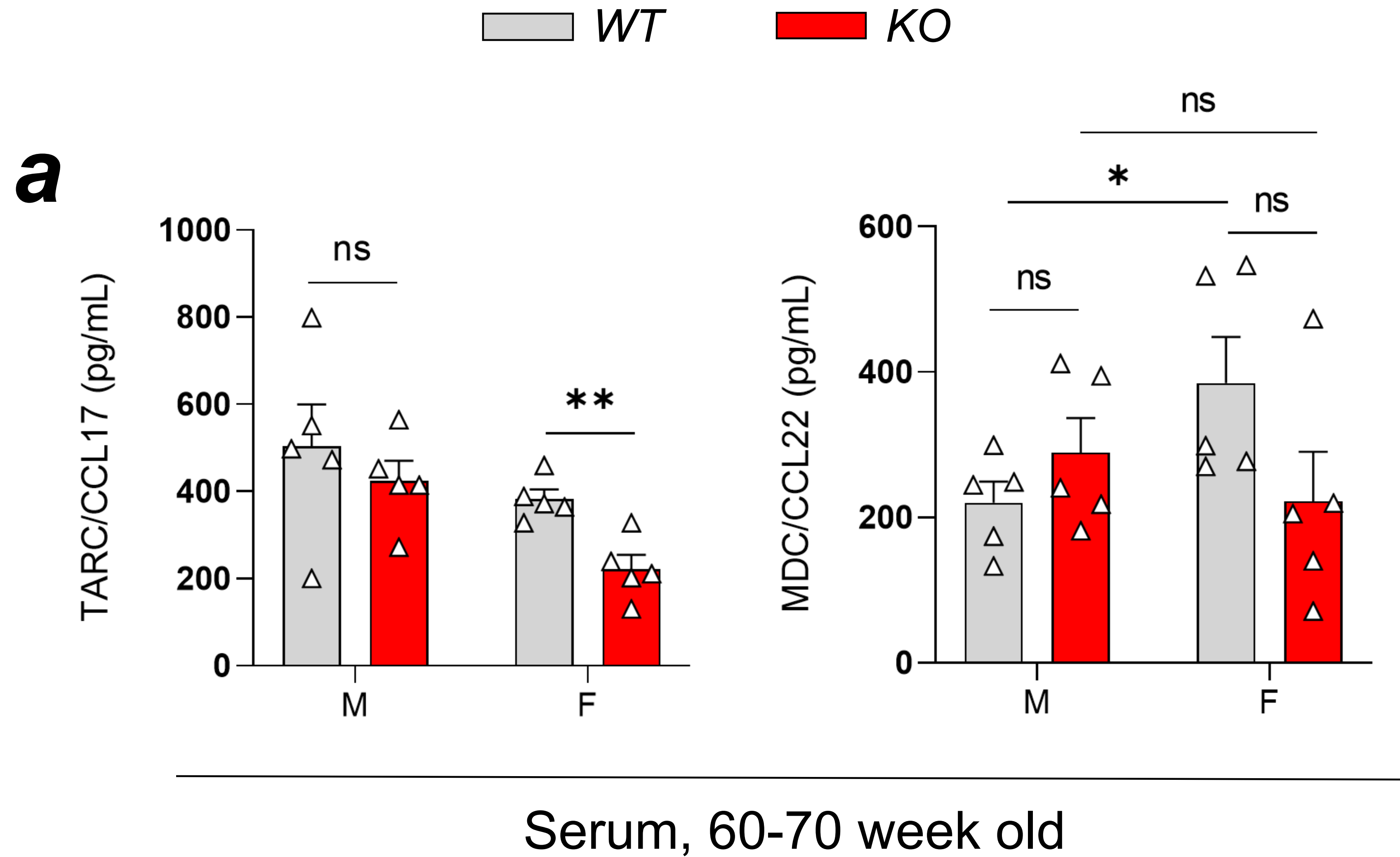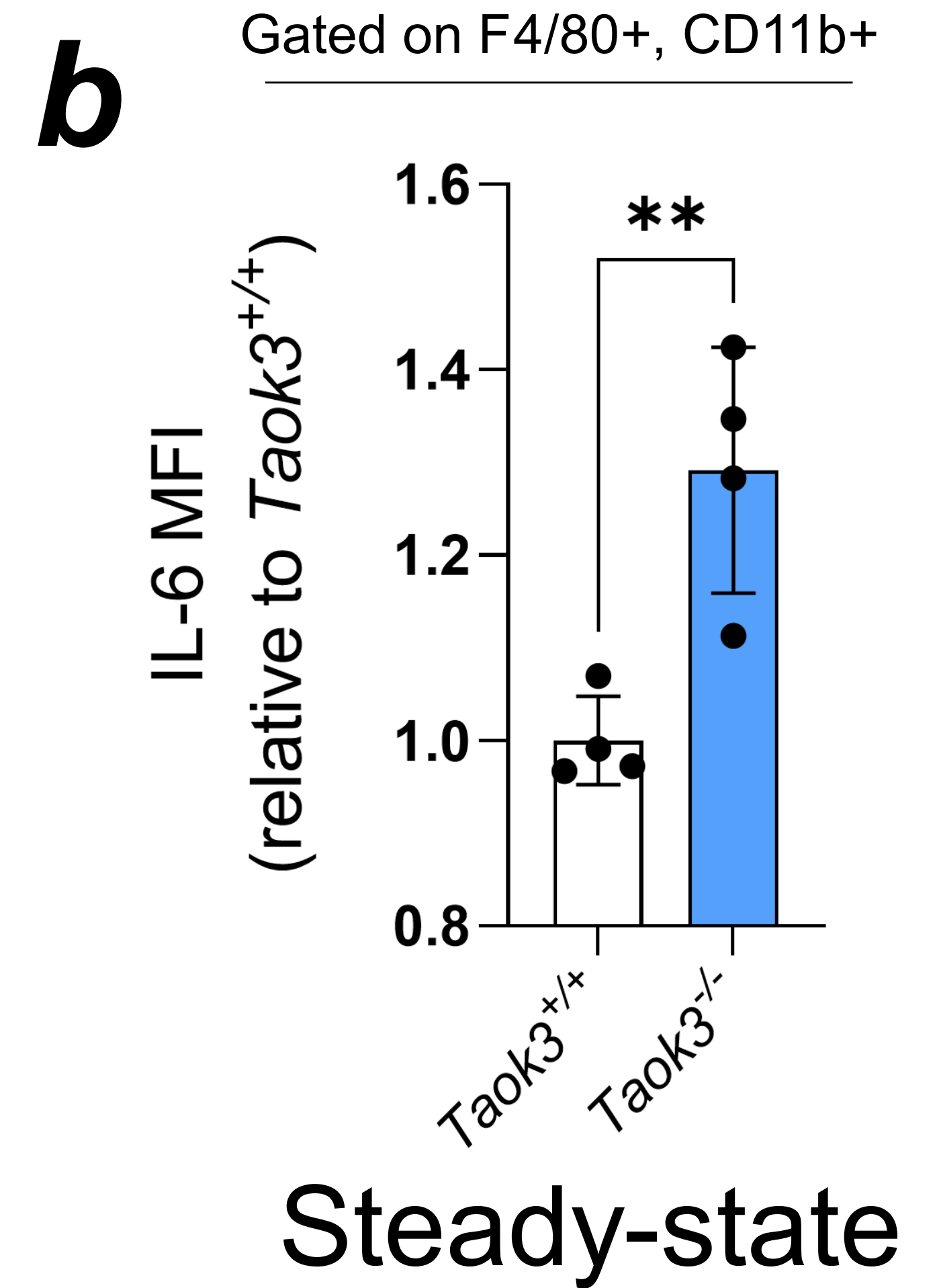

Supplement: Supplementary file 3 — Additional file 3: Sup. Fig. 3. Additional cytokine profiling in Taok3+/+ and Taok3-/- mice. a) Cytokine multiplexing quantification of TARC/CCL17 and MDC/CCL22 levels in serum of mice stratified by genotype and sex. b) IL-6 mean fluorescence expression in F4/80+, CD11b+ LPMs in steady state mice. Each animal represents one data point. Bar chart represents mean +/- SEM. Statistical analysis: (a-b) Multiple unpaired, bilateral T-tests. [file 12979_2023_350_MOESM3_ESM.pdf]
